# Changes in B-Cell Counts and Percentages during Primary HIV Infection Associated with Disease Progression in HIV-Infected Men Who Have Sex with Men: A Preliminary Study

**DOI:** 10.1155/2015/468194

**Published:** 2015-09-07

**Authors:** Chen Cui, Yongjun Jiang, Zining Zhang, Qinghai Hu, Zhenxing Chu, Junjie Xu, Bin Zhao, Haibo Ding, Jing Liu, Xiaoxu Han, Yaming Cao, Hong Shang

**Affiliations:** ^1^Key Laboratory of AIDS Immunology of the National Health and Family Planning Commission, Department of Laboratory Medicine, The First Affiliated Hospital, China Medical University, Shenyang 110001, China; ^2^Collaborative Innovation Center for Diagnosis and Treatment of Infectious Diseases, Hangzhou 310000, China; ^3^Department of Immunology, College of Basic Medical Sciences, China Medical University, Shenyang, Liaoning 110001, China

## Abstract

Numerous anomalies in B-cell phenotypes and functions have been described in HIV-infected individuals. However, the actual relationship between B cells and disease progression remains unclear. In this study, we investigated B-cell counts/percentages during a 12-month infection period in HIV-infected individuals that eventually developed into typical progressors (TPs) or rapid progressors (RPs). We found, after 12 months of infection, the baseline B-cell counts/percentages correlated positively with CD4^+^ T-cell counts (*P* = 0.0006 and *P* = 0.026) and negatively with HIV viral set points (*P* = 0.014 and *P* = 0.002). Kaplan-Meier survival analysis showed that high baseline B-cell counts/percentages were associated with a slow CD4-cell decline. B-cell kinetics indicated the baseline B-cell counts/percentages could be factors distinguishing between TPs and RPs. The combination of the baseline B-cell counts and percentages was associated with rapid disease progression (a 80.7% predictive value as measured by the area under the curve). These results indicate that the baseline B-cell counts/percentages might be associated with HIV disease progression.

## 1. Introduction

B cells play a vital role in the immune system, specifically in humoral immunity, which is a branch of the adaptive immune system. B cells can differentiate into plasma cells which secrete large amounts of antibodies to assist in the destruction of pathogens and infected cells. Activated B cells are full-time antigen-presenting cells (APCs), regulating T-cell functions via surface proteins such as CD40 and B7 and secreting various cytokines to participate in inflammatory responses and critical immunoregulation. Thus, anomalies in B-cell counts and functions may affect antiviral immune responses.

Acquired immunodeficiency syndrome (AIDS) is a human immune system disease caused by the human immunodeficiency virus (HIV). HIV infection is associated with abnormalities of all the major lymphocyte populations, including B cells. In 1983, B-cell hyperactivation and dysfunction were described in individuals with AIDS [1]. Following this, direct interactions between HIV and B cells were reported [2], and B-cell phenotypic alterations in HIV infection were also identified [3]. Further research revealed important aspects of the indirect effects of HIV viraemia on B cells; these included HIV-induced B-cell hyperactivity, HIV-induced lymphopenia, and HIV-associated B-cell exhaustion [4]. In addition, apoptotic mechanisms were described that might contribute to the progressive dysfunction and depletion of B cells in HIV disease [5].

In recent years, the pathogenic mechanisms of HIV-associated disease progression have been the subject of intense research. Mounting evidence has indicated that the immunological status of the patient in the early stages of HIV infection, in primary HIV infection (PHI), determines the subsequent progression of the disease [6]. However, in PHI subjects, the alterations in the absolute numbers of B cells and B-cell percentages of all leukocytes have not hitherto been adequately described. It has been reported that CD5^+^ B cells in HIV infection are related to HIV immunological progression [7] and that the percentages of memory B cells are correlated with CD4^+^ T-cell counts [8]. On this basis, we sought to gain a better understanding of the relationship between B cells in PHI and HIV disease progression by studying B-cell kinetics.

In almost every context studied, men who have sex with men (MSMs) are at substantial risk for HIV infection [9, 10]. In this population, certain factors, including known behavioural factors [11], can hasten the rate of disease transmission. In China, estimated 18 million men engage in homosexual activities, and HIV transmission rates between homosexuals continue to rise [12]. In addition, it has been reported that the declines in CD4 counts and increases in HIV-RNA are more rapid in Chinese MSMs compared to MSMs from high-income countries [13]. Therefore, further study is urgently needed on the impact of various factors relating to HIV disease progression among Chinese MSMs.

In this study, we examined B cells in a cohort of PHI-MSMs during their first 12-month follow-up period and compared the baseline counts of B cells during PHI with both CD4^+^ T-cell counts and viral loads at the time of the 12-month follow-up visit. We hoped to gain new insights into the role of B cells in HIV infection, changes in B-cell counts/percentages in relationship to CD4^+^ T cell lineage over the course of HIV infection, and the relationship between B-cell counts and HIV progression.

## 2. Material and Methods

### 2.1. Subjects

**Figure 1:**
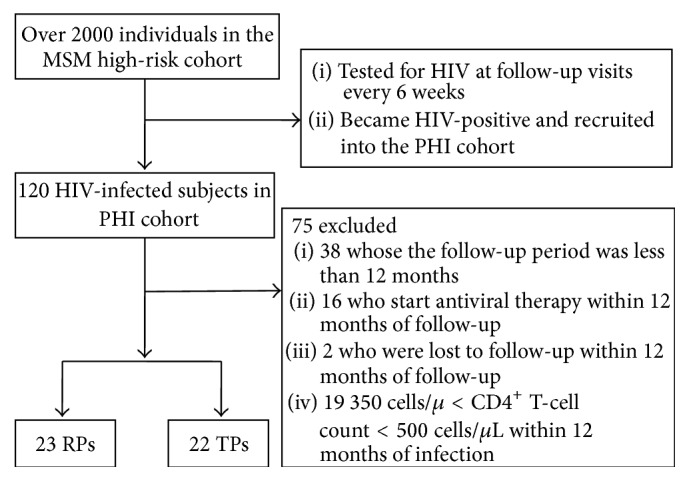
Procedure of sample selection. Over 2000 individuals were recruited and followed up in the MSM high-risk cohort. When they became HIV-positive, they were recruited into the PHI cohort. A total of 120 subjects were followed up in the PHI cohort. Clinical and laboratory measurements were taken as previously mentioned in Section 2.1. However, 75 did not meet the inclusion criteria. Finally, 23 RPs and 22 TPs were included in this study.

A total of 120 HIV-infected subjects with PHI were recruited from a high-risk MSM cohort in northeastern China. This high-risk MSM cohort of over 2000 individuals was openly and prospectively selected, via recruitment from HIV voluntary counselling and testing centres. Blood samples were obtained and tested for HIV at follow-up visits every 6 weeks. If the high-risk MSMs became HIV-positive, they were then excluded from the MSM high-risk cohort and recruited into the PHI cohort, providing they met the following criteria: (1) a detectable level of plasma HIV RNA and (2) an indeterminate Western blot. The PHI cohort was open and prospective. The date of infection was estimated on the basis of the patient's laboratory and epidemiological results, according to the Fiebig classification [14]. Subjects with CD4^+^ T-cell counts <350 cells/*μ*L during the study, or who were willing to undergo treatment, were offered antiviral therapy and removed from the PHI cohort. Hence, 23 rapid progressors (RPs) and 22 typical progressors (TPs) were finally selected for inclusion in this study.  Figure 1 showed the procedure of sample selection. The subjects were all MSMs of Han ethnicity, this being the predominant ethnic group in China (accounting for 91.51% of the population). RPs were defined as HIV-infected individuals who were treatment-naïve and had CD4^+^ T-cell counts <350 cells/*μ*L within 1 year of infection. TPs were treatment-naïve subjects whose CD4^+^ T-cell counts remained ⩾500 cells/*μ*L within 1 year of infection. Subjects' clinical and laboratory measurements were taken on study entry (baseline) and subsequently as follows: once a week during the first month, once a month for the second and third months, once every three months from the fourth to the 12th month, and after 1 year, once every six months. For comparison purposes, 24 healthy, HIV-negative individuals were recruited as a HIV-negative control group (“HIV-negative” in Table 1 and Supplementary Figure 1; see Supplementary Material available online at http://dx.doi.org/10.1155/2015/468194). The study protocol and the informed consent forms were both approved by the Ethical Review Board of the First Affiliated Hospital of China Medical University. Informed consent was obtained from all the study subjects.

### 2.2. Determination of B-Cell Absolute Counts

B-cell counts were measured by a FACSCalibur flow cytometer (BD Bioscience, San Jose, CA, USA). A single-platform lyse-no-wash procedure was performed using TruCOUNT tubes and MultiTEST CD3-fluorescein isothiocyanate (FITC) (clone SK7)/CD16+56-phycoerythrin (PE) (CD16, clone B73.1; CD56, clone NCAM 16.2)/CD45-peridinin chlorophyll protein (PerCP) (clone 2D1 (HLe-1))/CD19-allophycocyanin (APC) (clone SJ25C1) reagents (BD, USA). The fluorescent beads in the BD TruCOUNT tubes were the internal control. The samples were then analysed by flow cytometry with the FACS MULTISET software to count B cells.

### 2.3. Determination of CD4^+^ T-Cell Absolute Counts

CD4^+^ T-cell counts were measured by a FACSCalibur flow cytometer (BD Bioscience, San Jose, CA, USA). A single-platform lyse-no-wash procedure was performed using TruCOUNT tubes and TriTEST anti-CD4-FITC (clone SK3)/CD8-PE (clone SK1)/CD3-PerCP (clone SK7) reagents (BD, USA). The fluorescent beads in the BD TruCOUNT tubes were the internal control. TruCOUNT control beads (low, median, and high beads) were used to ensure the quality of CD4^+^ T-cell test.

### 2.4. HIV Viral Load Measurement

HIV viral loads in plasma were detected by RT-PCR with the COBAS Amplicor HIV Monitor 1.5 (Roche Molecular Systems, Branchbury, NJ, USA). A detection limit was between 400 copies/mL and 7.5 × 10^5^ copies/mL.

### 2.5. Statistical Analysis

Log_10_-transformed values of plasma HIV RNA levels were used. Categorical data were described and analysed by frequency and chi-square (*χ* ^2^) test. Kolmogorov-Smirnov test (50 ⩽ sample sizes ⩽ 1000) and Shapiro-Wilk test (3 ⩽ sample sizes ⩽ 50) were used to test if the variables were normal distributed. Independent *t*-test and one-way ANOVA were used to analyse the normally distributed continuous variables between 2 groups and among 3 groups, respectively. The Mann-Whitney *U* test was used to analyse the nonnormally distributed continuous variables. Spearman's rank correlation or Pearson correlation was used to perform the correlation analysis. Kaplan-Meier survival analysis was used to examine the effects of B-cell counts and percentages on HIV disease progression. Loess curve fitting was used to describe the changes of the parameters in HIV-infected individuals under the estimated infection time and was plotted with SPSS 17.0 software. All analyses were carried out using SPSS 17.0 software. *P* values < 0.05 were considered statistically significant. Receiver-operating characteristic (ROC) curves and the area under the ROC curve (AUC) were used to assess the sensitivity and specificity of the baseline B-cell counts and percentages for the predictive performance of HIV disease progression. For ROC analysis of the combination for the baseline B-cell count and percentage, *P* (probability of a patient sample) was calculated for inclusion in the ROC analysis by the following formula: *X* = logit⁡(*P*) = ln⁡(*P*/1 − *P*) = *b*_0_ + *b*_1_ ×  the baseline B-cell counts + *b*_2_  ×  the baseline B-cell percentages. In this study, *X* = logit⁡(*P*) = 2.454 − 0.021  ×  the baseline B-cell counts + 0.132 × the baseline B-cell percentages; *P* = *e* ^*x*^/(1 + *e* ^*x*^).

## 3. Results

### 3.1. Subject Characteristics

Demographic and baseline immunological comparisons between the 120 HIV-infected subjects with PHI and the 24 HIV-negative individuals are summarised in Table 1. We found that the baseline parameters including B-cell counts, B-cell percentages, and CD4^+^ T-cell counts in HIV-infected MSMs were significantly lower than those in the healthy HIV-negative individuals (*P* < 0.001 for all). However, the baseline CD8^+^ T-cell median counts in HIV-infected MSMs were much higher than those in the healthy HIV-negative individuals (*P* < 0.001).

**Figure 2:**
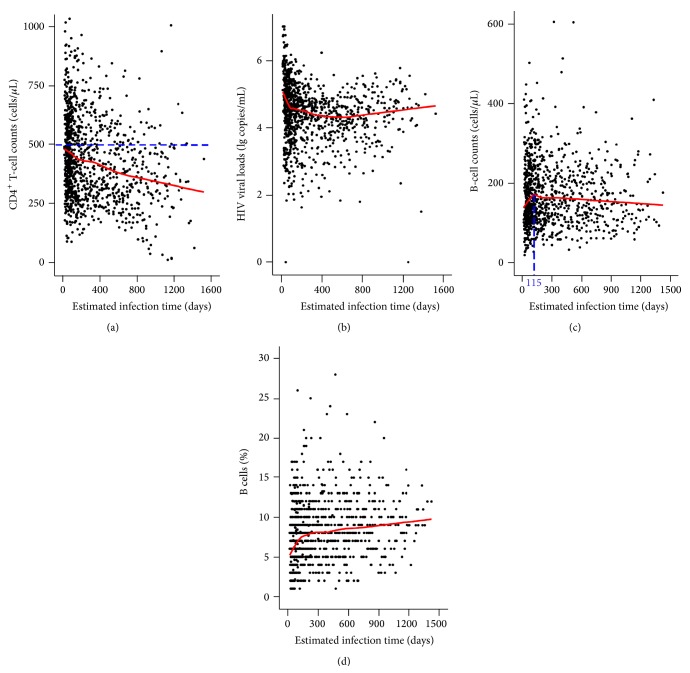
Changes of the parameters over time in HIV-infected individuals. Loess curve fitting was applied to this study and performed with SPSS 17.0 software. Changes in the parameters, including CD4^+^ T-cell counts (a), HIV viral loads (b), B-cell counts (c), and percentages of B cells (d), in 120 HIV-infected subjects were plotted. Note also (a) CD4^+^ T-cell counts declined after HIV seroconversion. The solid line (red) is the curve obtained from Loess fitting. The dotted line (blue) represents the level of CD4^+^ T-cell counts for the majority of healthy HIV-negative individuals (500 cells/*μ*L). (b) After HIV infection, HIV viral loads underwent a rapid decline and then entered a stable state before starting a slow increase. The solid line (red) is the curve obtained from Loess fitting. (c) B-cell counts showed a rapid recovery and then, at approximately 115 days (blue dotted line), began to slowly decline after HIV seroconversion. The solid line (red) is the curve obtained from Loess fitting. (d) B-cell percentages experienced a gradual recovery. The solid line (red) is the curve obtained from Loess fitting.

As previously described in Section 2.1, all parameters of the PHI-cohort participants were examined during each visit. To better characterise the 120 HIV-infected subjects with PHI, the parameter dynamics, including CD4^+^ T-cell counts, HIV viral loads, B-cell counts, and B-cell percentages, are depicted in Figure 2. Loess curve fitting was applied to this study. We found that CD4^+^ T-cell counts declined in PHI subjects (Figure 2(a)). During the early phases of HIV infection, plasma viral loads dropped rapidly within months, reached a relatively stable level, and then underwent a slow increase over the period of chronic infection (Figure 2(b)). B-cell counts recovered rapidly and began to slowly decline from approximately 115 days after HIV seroconversion (Figure 2(c)). However, the percentages of B cells underwent a gradual recovery (Figure 2(d)).

To explore the association between absolute counts and percentages of B cells in primary HIV infection and throughout the disease progression, 45 HIV-infected MSMs (23 RPs and 22 TPs) who satisfied the criteria were included in our continuing study. The RPs and TPs were similar in age, HIV subtype (CRF01_AE), estimated duration of infection at study entry, and length of follow-up. The baseline HIV viral loads and HIV viral loads after 12 months of the infection were higher in RPs than in TPs, but the difference was not statistically significant (*P* = 0.670 and *P* = 0.220, resp.) (data not shown). However, in RPs the baseline CD4^+^ T-cell counts and CD4^+^ T-cell counts after 12 months of HIV infection were significantly lower than those in TPs (*P* < 0.001 and *P* < 0.001, resp.).

### 3.2. B-Cell Counts in RPs and TPs at the Baseline Visit and the 12-Month Follow-Up Visit

We found that the baseline B-cell counts were lower in RPs compared with TPs and in HIV-negative volunteers (*P* = 0.001 and *P* = 0.034, resp.; Supplementary Figure 1(a)). The baseline B-cell percentages were also lower in RPs than in HIV-negative volunteers (*P* < 0.001; Supplementary Figure 1(c)). Furthermore, at the 12-month follow-up visit, B-cell counts of RPs were significantly lower than those of TPs and HIV-negative volunteers (*P* < 0.001 and *P* < 0.001, resp.; Supplementary Figure 1(b)). In addition, B-cell percentages of RPs at the 12-month follow-up visit were lower than those of HIV-negative volunteers (*P* = 0.011; Supplementary Figure 1(d)).

### 3.3. Comparison of CD4^+^ T-Cell Counts and HIV Viral Loads at the Baseline Visit and 12-Month Follow-Up Visit between Different Groups Based on the Baseline B-Cell Counts

CD4^+^ T cells and viral loads are recognised as the primary markers of immunodeficiency in HIV infection. To better investigate the association between B-cell counts and disease progression, we studied the effects of the baseline B-cell counts and percentages on CD4^+^ T cells and viral loads. First, we grouped together 45 HIV-infected subjects based on their absolute B-cell counts or percentages at the baseline visit. Subjects were put into the “B < median” group when their absolute baseline B-cell counts were below the median (139 cells/*μ*L). Alternatively, subjects were put into the “B ⩾ median” group when their absolute baseline B-cell counts were above or equal to the median. Similarly, our study subjects were also grouped into the “Percent B < median” and the “Percent B ⩾ median” group based on subjects' baseline B-cell percentages when compared to the median (7%).

Then, we compared CD4^+^ T-cell counts and viral loads at the baseline visit between the aforementioned groups. We found that CD4^+^ T-cell counts were much higher in the “B ⩾ median” group (*n* = 23) than in the “B < median” group (*n* = 22) (*P* = 0.0002; Supplementary Figure 2(a)). However, viral loads were lower in the “Percent B ⩾ median” group (*n* = 23) than in the “Percent B < median” group (*n* = 22) (*P* = 0.002; Supplementary Figure 2(b)).

Next, CD4^+^ T-cell counts and HIV viral loads between the aforementioned groups were compared at their 12-month follow-up visits. We found that, after 12 months of HIV infection, CD4^+^ T-cell counts remained higher in the “B ⩾ median” group than in the “B < median” group (*P* = 0.0047; Supplementary Figure 2(c)). However, there was no statistical difference between the viral loads of the different groups (Supplementary Figure 2(d)).

Overall, these findings indicate that the high absolute B-cell counts might be associated with the maintenance of high CD4^+^ T-cell counts.

### 3.4. Correlations of the Baseline B-Cell Counts and Percentages with CD4^+^ T-Cell Counts and HIV Viral Loads

Subjects who progressed to RPs or TPs exhibited distinct differences in their B-cell counts when compared to both HIV-negative individuals and to each other. Hence, we decided to further investigate the potential relationship between the counts/percentages of B cells at the time of initial infection, and CD4^+^ T-cell counts/viral loads at the baseline visit plus the 12-month follow-up visit.

**Figure 3:**
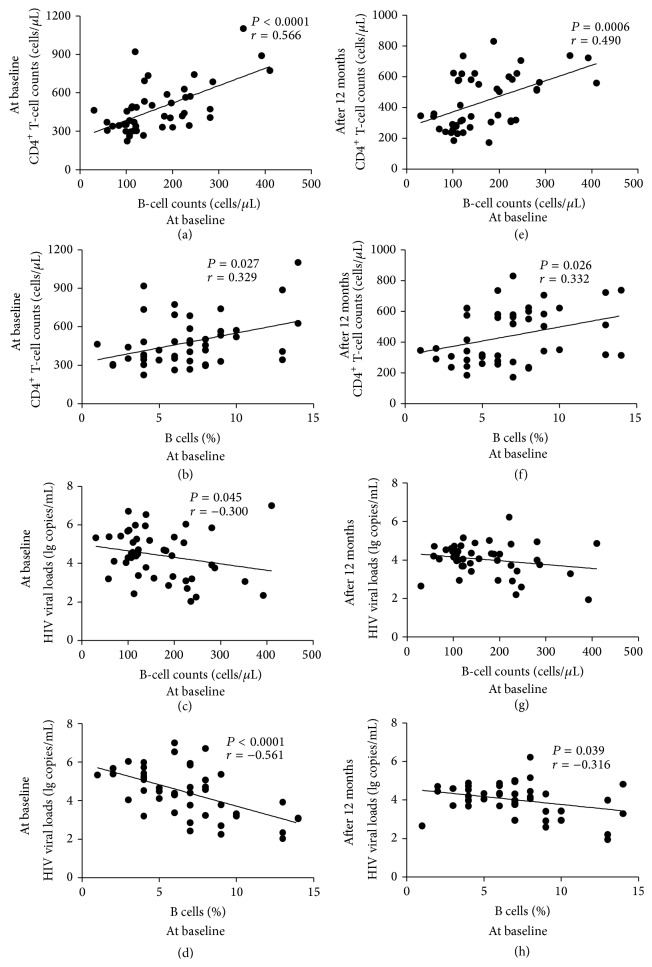
Correlation between B-cell counts/percentages and CD4^+^ T-cell counts/HIV viral loads in HIV-infected individuals. The correlations between the baseline B-cell counts and CD4^+^ T-cell counts (a) at the baseline and (e) after 12 months. The correlations between the baseline percentages of B cells and CD4^+^ T-cell counts (b) at the baseline and (f) after 12 months. The correlations between the baseline B-cell counts and HIV viral loads (c) at the baseline and (g) after 12 months. The correlations between the baseline percentages of B cells and HIV viral loads (d) at the baseline and (h) after 12 months. *P* values < 0.05 are considered statistically significant.

The baseline counts of B cells showed a positive correlation with the baseline CD4^+^ T-cell counts (*P* < 0.0001, *r* = 0.566; Figure 3(a)) and a negative correlation with baseline HIV viral loads (*P* = 0.045, *r* = −0.300; Figure 3(c)). Additionally, the baseline percentages of B cells correlated positively with the baseline CD4^+^ T-cell counts (*P* = 0.027, *r* = 0.329; Figure 3(b)) and negatively with the baseline viral loads (*P* < 0.0001, *r* = −0.561; Figure 3(d)). Furthermore, the baseline counts and percentages of B cells correlated positively with CD4^+^ T-cell counts even after 12 months of HIV infection (*P* = 0.0006, *r* = 0.490, and *P* = 0.026, *r* = 0.332, resp.; Figures 3(e) and 3(f)). No correlations were found between B-cell counts at the baseline visit and HIV viral loads at the 12-month follow-up visit (*P* = 0.187, *r* = −0.205; Figure 3(g)). However, the baseline B-cell percentages were negatively correlated with HIV viral loads at the 12-month follow-up visit (*P* = 0.039, *r* = −0.316; Figure 3(h)).

### 3.5. CD8^+^ T-Cell Counts/Percentages Might Be Weak Predictors of the Disease Progression

From these results, we concluded that the baseline B-cell counts might be associated with HIV disease. Our investigation of B cells and their role in humoral immunity led us to consider CD8^+^ T cells, as these cells play a significant role in cellular immunity. Therefore, we conducted an experiment to study the effect of baseline CD8^+^ T-cell counts on HIV disease progression. Initially, we grouped our 45 study subjects according to how their baseline CD8^+^ T-cell counts or percentages compared with the median values. The baseline CD8^+^ T-cell median count and median percentage were 1095 cells/*μ*L and 73%, respectively. According to these criteria, 23 subjects whose baseline CD8^+^ T-cell counts were above 1095 cells/*μ*L were assigned to the “CD8 ⩾ median” group, and 22 subjects whose baseline CD8^+^ T-cell counts were below 1095 cells/*μ*L were assigned to the “CD8 < median” group. Among these individuals, 24 subjects whose baseline CD8^+^ T-cell percentages were greater than 73% were placed in the “CD8% ⩾ median” group, and 21 subjects whose baseline CD8^+^ T-cell percentages were less than 73% were placed in the “CD8% < median” group. Firstly, CD4^+^ T-cell counts and viral loads at the baseline visit and the 12-month follow-up visit were compared between the groups of “CD8 ⩾ median” or “CD8 < median” and “CD8% ⩾ median” or “CD8% < median.” The CD4^+^ T-cell counts at the baseline were found to be lower in the “CD8% ⩾ median” group than in the “CD8% < median” group (*P* = 0.0015; Supplementary Figure 3(a)), and CD4^+^ T-cell counts at the 12-month follow-up visit remained lower in the “CD8% ⩾ median” group than in the “CD8% < median” group (*P* = 0.0007; Supplementary Figure 3(b)). The baseline viral loads were higher in the “CD8 ⩾ median” and “CD8% ⩾ median” groups than in the “CD8 < median” and “CD8% < median” groups (*P* = 0.015 and *P* < 0.001; Supplementary Figure 3(a)). In addition, viral loads at the 12-month follow-up visit were higher in the “CD8% ⩾ median” group than in the “CD8% < media” group (*P* = 0.009; Supplementary Figure 3(b)).

Secondly, measurements were taken of the correlation of the baseline counts/percentages of CD8^+^ T cells with CD4^+^ T-cell counts or viral loads at the baseline visit or the 12-month follow-up visit. The baseline percentages of CD8^+^ T cells correlated negatively with both CD4^+^ T-cell counts at the baseline visit and the 12-month follow-up visit (*P* = 0.0002, *r* = −0.531 and *P* = 0.0002, *r* = −0.530, resp.; Supplementary Figures 3(c) and 3(d)). However, the baseline percentages of CD8^+^ T cells correlated positively with viral loads both at the baseline visit and the 12-month follow-up visit (*P* < 0.0001, *r* = 0.556, and *P* = 0.02, *r* = 0.346, resp.; Supplementary Figures 3(c) and 3(d)). Based on these findings, we concluded that the baseline CD8^+^ T-cell counts/percentages might be weak predictors of disease progression.

### 3.6. Association of the Baseline B-Cell Counts/Percentages with HIV Viral Set Point

**Figure 4:**
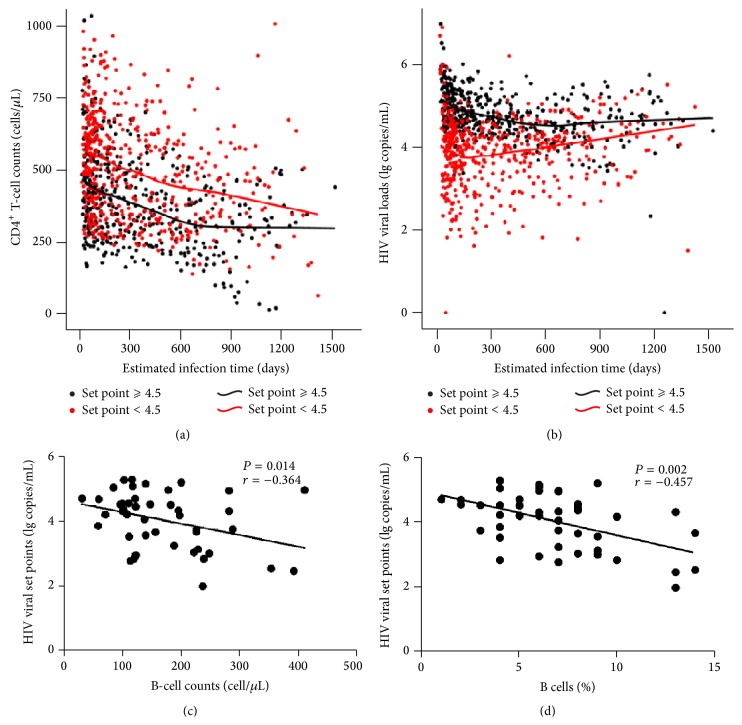
Correlation between B-cell counts/percentages at the baseline visit and HIV viral set point. Using data from the 120 PHI subjects, changes in the CD4^+^ T cells (a) and viral loads (b) over time were plotted. Loess curve fitting was applied to the data and performed with SPSS 17.0 software. By comparison with those individuals whose viral set points were below 4.5 lg copies/mL (red), the individuals whose viral set points were above or equal to 4.5 lg copies/mL (black) had persistently lower CD4^+^ T-cell counts but higher viral loads. (c) Using data from the 45 subjects, the baseline B-cell counts were correlated negatively with HIV viral set points (*P* = 0.015, *r* = −0.402). (d) The percentages of B cells at the baseline were correlated with HIV viral set points (*P* = 0.008, *r* = −0.435). *P* values < 0.05 are considered statistically significant.

It has been shown that the initial viral set point after primary HIV infection is a very strong predictor of the duration of the disease-free period until the onset of AIDS [15–17]. It has been reported that HIV-infected individuals with a higher viral set point progress faster to the onset of AIDS and to death [18]. Therefore, we explored the association between the baseline B-cell counts/percentages and the HIV viral set point. To maximise accuracy, the HIV viral set point was defined as the mean of viral load measurements taken at 3 time points between 120 days and 1 year following the initial HIV infection. Among the 120 PHI subjects, we observed that those subjects whose viral set points were above or equal to 4.5 lg copies/mL had persistently lower CD4^+^ T-cell counts and higher viral loads throughout the course of their infection compared to those whose viral set points were below 4.5 lg copies/mL (Figures 4(a) and 4(b)). In addition, we sought to determine whether the baseline B-cell counts or percentages had any association with HIV viral set points. Our results showed that in 45 subjects, baseline B-cell counts correlated negatively with HIV viral set points (*P* = 0.014, *r* = −0.364; Figure 4(c)). Similarly, the baseline percentages of B cells showed a significant negative-correlation with HIV viral set points (*P* = 0.002, *r* = −0.457; Figure 4(d)). These correlations indicated that the baseline B-cell percentages/counts might be associated with HIV disease progression.

### 3.7. Survival Rates Significantly Decreased in HIV-Infected Subjects with Low Baseline B-Cell Counts and Percentages

To further confirm the possible association between the baseline B-cell counts and HIV disease progression, we carried out a survival analysis to determine the effects of the baseline B-cell counts. Using the median baseline B-cell count (139 cells/*μ*L) as a cutoff, we separated the 45 HIV-infected MSMs into the “B ⩾ median” group and the “B < median” group. Endpoint events were considered to be CD4^+^ T-cell counts dropping below 350 cells/*μ*L or HIV viral loads reaching 10^4.5^ copies/mL.

**Figure 5:**
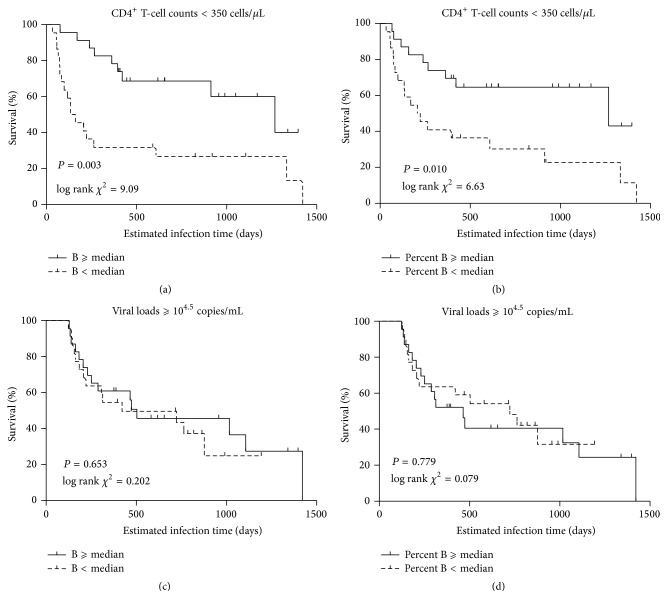
The effect of the baseline B-cell counts/percentages on HIV disease progression. According to the median value of the baseline B-cell counts/percentages, 45 study participants were divided into the “B ⩾ median” group/the “Percent B ⩾ median” group (above the median value) and the “B < median” group/the “Percent B < median” group (below the median value). Kaplan-Meier survival analysis was used to separately compare the baseline B-cell counts (a, c) and percentages (b, d) regarding disease progression. CD4^+^ T-cell counts < 350 cells/*μ*L (a, b) and HIV viral load ⩾ 10^4.5^ copies/mL (c, d) were considered as the endpoint events. *P* values < 0.05 are considered statistically significant.

Kaplan-Meier survival analysis showed that in subjects with lower baseline B-cell counts the mean amount of time needed for CD4^+^ T-cell counts to drop to below 350 cells/*μ*L was significantly shorter than in those subjects with higher baseline B-cell counts (log rank *χ* ^2^ = 9.09, *P* = 0.003; Figure 5(a)). Low baseline B-cell counts seemed to have no effect on the amount of time needed for viral loads to reach 10^4.5^ copies/mL (log rank *χ* ^2^ = 0.202, *P* = 0.653; Figure 5(c)). This survival analysis showed that HIV-infected MSMs with low baseline B-cell counts during PHI were more likely to experience a much more rapid decline in their CD4^+^ T-cell counts.

Additionally, we investigated whether low baseline B-cell percentages were a predictor of rapid HIV disease progression. In this survival analysis, lower baseline B-cell percentages predicted a faster CD4^+^ T-cell decline (log rank *χ* ^2^ = 6.63, *P* = 0.010; Figure 5(b)). However, the baseline B-cell percentages did not predict the increase of the viral-load rate (log rank *χ* ^2^ = 0.079, *P* = 0.779; Figure 5(d)).

### 3.8. CD4^+^ T-Cell Counts and Viral Loads Were Associated with Baseline B-Cell Counts and Percentages

**Figure 6:**
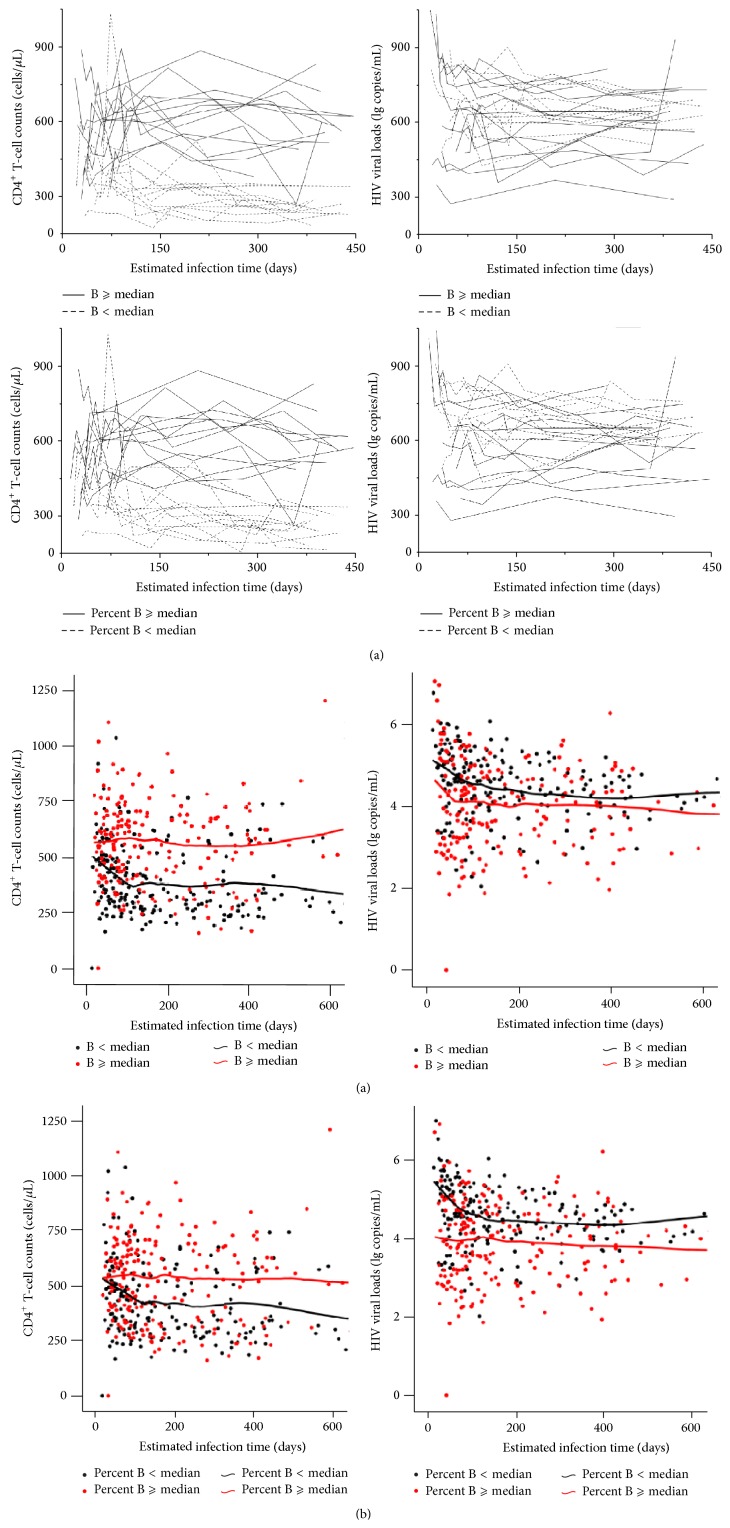
Persistent elevation in CD4^+^ T-cell counts and persistent low-level of viral loads of the individuals with higher baseline B-cell counts. We grouped 45 HIV-infected subjects based on the absolute B-cell counts or percentages at the baseline visit. Subjects were put into the “B < median” group when their absolute baseline B-cell counts were below the median (139 cells/*μ*L). In contrast, subjects were put into the “B ⩾ median” group when their absolute baseline B-cell counts were above or equal to the median. Similarly, our study subjects were also grouped into the “Percent B < median” group and the “Percent B ⩾ median” group based on how subjects' baseline B-cell percentages compared to the median (7%). (a) The changes of CD4^+^ T-cell counts or HIV viral loads were plotted. The rapid drop in CD4^+^ T-cell counts and persistently higher viral loads were shown in the individuals with the low baseline B-cell counts and percentages. Each line represents 1 individual. Each solid line (—) represents one individual in the “B *⩾* median” group or the “Percent B ⩾ median” group. Each dotted line (-* *-* *-) represents one individual in the “B < median” group or the “Percent B < median” group. (b) The changes of the CD4^+^ T-cell counts and HIV viral loads were plotted the same as in Figure 6(a). Loess curve fitting was applied to the data. By comparing with the “B < median” group and the “Percent B < median” group (blank), CD4^+^ T-cell counts were elevated in the “B ⩾ median” group and the “Percent B ⩾ median” group (red).

In our study of HIV-infected MSMs in PHI, we monitored longitudinal changes in CD4^+^ T-cell counts and viral loads over the course of the first 12 months of untreated infection. As previously mentioned in Section 3.3, we divided our 45 subjects into groups based on two classifications as follows: (1) “B ⩾ median” and “B < median,” and (2) “Percent B ⩾ median” and “Percent B < median.” Next, we plotted the dynamic progression of the CD4^+^ T-cell counts and viral loads for each subject at several time points. During the first 12 months of infection, subjects with higher baseline B-cell counts had persistently high CD4^+^ T-cell counts and low viral loads (Figure 6(a)). In addition, CD4^+^ T-cell counts remained higher in subjects with higher baseline B-cell percentages than in those with lower baseline B-cell percentages (Figure 6(a)). Likewise, HIV viral loads remained lower in subjects with higher baseline B-cell percentages than in those with lower baseline B-cell percentages (Figure 6(a)).

To describe the changes of the CD4^+^ T-cell counts and viral loads within the groups, Loess curve fitting was applied. We found that subjects in the “B ⩾ median” group or the “Percent B ⩾ median” group had persistently higher CD4^+^ T-cell counts and lower viral loads than those in the “B < median” group or the “Percent B < median” group (Figure 6(b)).

Overall, these data show that CD4^+^ T-cell counts and viral loads are significantly associated with baseline B-cell counts and percentages, which indicates that early events in the host-pathogen interactions might determine the progression of the HIV disease.

### 3.9. B-Cell Counts and Percentages in PHI Might Distinguish TPs from RPs

**Figure 7:**
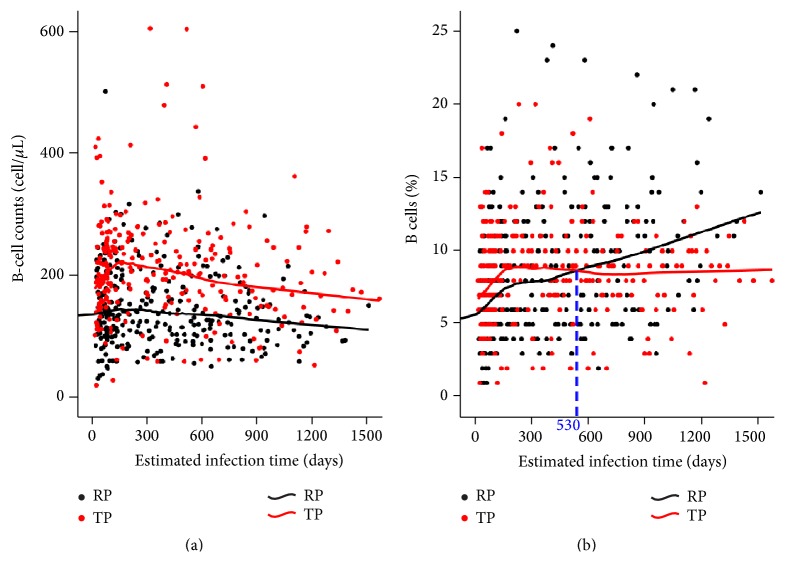
Longitudinal changes of the B-cell counts and percentages in TPs and RPs. Loess curve fitting was applied to this study and performed with SPSS 17.0 software. (a) During HIV infection, TPs (red) in our study had persistently higher B-cell counts than RPs (black). (b) Within 530 days of infection (blue dotted line), TPs had higher B-cell percentages than RPs. Then, B-cell percentages of TPs (red) dropped below those of RPs (black). Loess curve fitting was applied to the study.

Since our findings indicated that B-cell counts and percentages in PHI might affect disease progression, we therefore compared the longitudinal changes in B-cell counts and percentages between TPs and RPs. We found that TPs had persistently higher B-cell counts than RPs did (Figure 7(a)). Up until 530 days after infection, TPs in our study had higher B-cell percentages than RPs did. However, after 530 days, B-cell percentages in TPs were lower than those in RPs (Figure 7(b)). This suggests that B-cell counts and percentages in PHI could be another characteristic distinguishing TPs from RPs in HIV-infected individuals.

### 3.10. Baseline B-Cell Counts and Percentages Were Associated with Disease Progression

**Figure 8:**
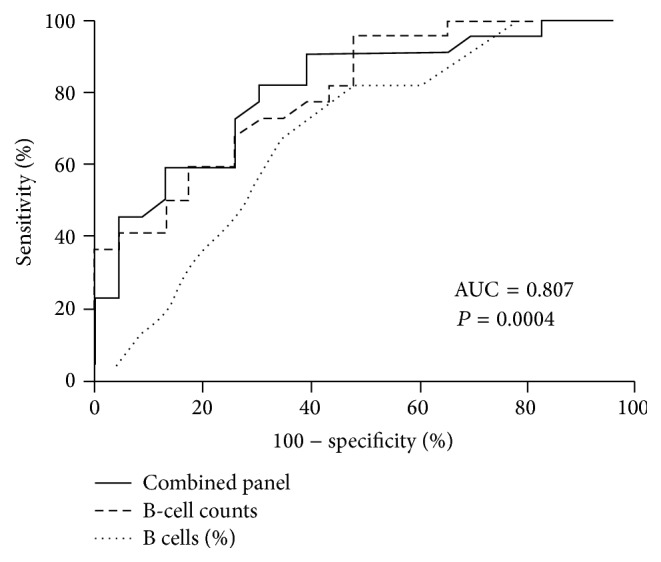
The baseline B-cell counts and percentages were associated with the disease progression. The predictive values of the baseline B-cell counts, percentages, and the predictive combination of the two for rapid disease progression were calculated by ROC analysis. The area under the curve (AUC) was used to predict disease progression. *P* value < 0.05 is considered statistically significant.

Finally, we examined whether the baseline B-cell counts and percentages that differentiated RPs from TPs were related to the disease progression. Using ROC analysis, we calculated the predictive power (AUC, area under the curve) of the baseline B-cell counts and percentages for disease progression. The combined panel of the baseline B-cell count with percentages showed an increased estimated AUC value when compared to the baseline B-cell counts or baseline B-cell percentages. The ROC curves had AUCs of 80.2% for the baseline B-cell counts (95% CI, 67.7%–92.8%) and 68.6% for the baseline B-cell percentages (95% CI, 52.8%–84.3%). In comparison, the AUC for the combined panel of the baseline B-cell counts and percentages was 80.7% (95% CI, 68.0%–93.53%) (*P* = 0.0005; Figure 8).

## 4. Discussion

This study showed that B-cell counts and percentages of HIV-infected MSMs during PHI are linked to HIV disease progression and the decline of CD4^+^ T-cell counts. Subjects who had higher baseline B-cell counts and percentages had persistently higher CD4^+^ T-cell counts and relatively low viral loads. More importantly, we found in our longitudinal study that subjects who had higher CD4^+^ T-cell counts throughout the infection period and had slow disease progression had persistently higher B-cell counts. The association between T and B cells was consistent when viewed from the perspective of either B- or T-cell counts. Moreover, by using the baseline B-cell counts and percentages we have developed a model to predict HIV disease progression with an AUC of 80.7%.

HIV infection progresses through a number of different phases. During the period of primary infection, viral replication increases and the immune system mounts a response in an attempt to control the viraemia. The subsequent depletion of CD4^+^ T cells and the increase in HIV viral loads lead to an impairment of the entire immune system. Finally, opportunistic infections occur, leading to AIDS and often finally to death [19–21]. Focusing on the earliest event in the progression of HIV disease, we studied an open prospective cohort of 120 subjects with PHI, recruited from a cohort of over 2000 MSMs at high risk for HIV infection. Following infection with HIV, both B-cell counts and percentages declined in all subjects, but these parameters displayed different patterns of change. B-cell counts underwent a rapid recovery and then began to decline slowly from approximately 115 days after HIV seroconversion. However, the percentages of B cells recovered gradually. We therefore speculate that, in the early phases of HIV infection, the virus stimulates reactive expansion of B cells. In particular, as the infection progressed, B-cell counts did not decline as quickly as other populations of lymphocytes did and the percentage of B cells of all lymphocytes underwent a gradual recovery.

During the clinical latency period, there are large variations in the response of treatment-naïve HIV-infected individuals, resulting in different disease progression rates. This individual variability suggests that there are host factors associated with HIV progression. Significantly decreased B-cell counts of RPs were observed at both the baseline and the 12-month follow-up visits as compared to the B-cell counts of TPs and healthy HIV-negative subjects (*P* = 0.001, *P* < 0.001, *P* < 0.001, and *P* < 0.001, resp.). The differences suggest that HIV infection might result in a decrease of B cells, and, therefore, the values of the baseline B-cell counts might be associated with the disease progression. However, we also found that the correlations in the figures were low. We considered that besides B cells, many other factors such as NK cells and plasmacytoid dendritic cells have also been reported to be associated with HIV disease progression [22, 23].

The depletion of CD4^+^ T cells is a hallmark of HIV disease progression [24], which makes CD4^+^ T-cell counts a powerful predictor of the short-term risk of progression to AIDS [25]. In our study, we observed that baseline B-cell counts correlated positively with CD4^+^ T-cell counts both at the baseline and at the 12-month follow-up visits. In the survival analysis and the longitudinal study, low baseline B-cell counts had an obvious influence on accelerating both the decline of CD4^+^ T cells and the increase of viral loads. These findings suggest that the baseline B-cell counts might be associated with the disease progression and especially in the decline of CD4^+^ T cells. Several mechanisms have been proposed for the interactions between B cells and CD4^+^ T cells [26–29]. B cells participate in inducing primary T-cell proliferation and generating, maintaining, and reactivating TH1 and TH2 memory CD4^+^ T cells via an antigen-specific but antibody-independent mechanism [26]. Additionally, in a study of lymphocytic choriomeningitis virus (LCMV) infection [27], it has been shown that the generation of CD4^+^ T-cell memory requires B cells. Therefore, we can infer that B cells might play a role in CD4^+^ T-cell memory during HIV infection. Müller et al., 1998 [28], found a significant correlation between serum immunoglobulin (Ig) levels and the percentage of CD4^+^ lymphocytes expressing CD40L in patients with HIV infection. De Milito et al., 2004 [29], observed that the capacity of patients' B cells to release immunoglobulins in vitro was correlated with their CD4^+^ T-cell counts, indicating that CD4^+^ T cells might play a crucial role in driving abnormal B-cell differentiation in HIV infection [29]. Furthermore, we speculate that this drop in number may indicate the bone marrow is not replenishing the peripheral B-cell pool during early HIV infection. The peripheral B-cell dysfunction or the decrease in counts during early HIV infection may affect CD4^+^ T-cell function and determine the subsequent disease progression.

Mellors et al., 1997 [30], have reported that plasma viral loads predicted the rate of decrease in CD4^+^ T-cell counts and progression to AIDS and death. In our study, we found that the baseline B-cell count and percentages were associated with HIV viral loads, both at the baseline visit and at the 12-month follow-up visit. However, the baseline CD8^+^ T-cell counts correlated positively with the baseline viral loads but did not correlate with viral loads at the 12-month follow-up visit. These correlations might be due to the large amount of HIV present in the early stages of infection, stimulating faster proliferation of CD8^+^ T cells. In addition, B cells secrete antibodies and target extracellular viruses, whereas CD8^+^ T cells mainly kill infected cells. The data indicate that the baseline CD8^+^ T-cell counts might be a weak predictor of the disease progression. In addition, we observed that B-cell counts and percentages at the baseline visit correlated negatively with HIV viral set points. We speculate that certain relationships exist between B cells and HIV viral loads. Multiple published studies have reported that CCR5 (HIV coreceptor) binding chemokines including macrophage inflammatory protein-*α* (MIP-*α*) and macrophage inflammatory protein-*β* (MIP-*β*), which are secreted by T cells, can suppress HIV infection [31–35]. Interestingly, a recent study has indicated that TLR-activated B cells secreted chemokines MIP-*α* and MIP-*β* as well [36]. In the PHI cohort of our study, most HIV-infected MSMs were infected with the R5-tropic virus. Therefore, we hypothesised that TLR-activated B cells might contribute to the disease suppression by the secretion of MIP-*α* and MIP-*β* during HIV infection, though this has not yet been proved. In a study by Moir et al., 2003 [37], B-cell proliferation correlated with CD40L expression on activated CD4^+^ T cells, whereas reduced B-cell proliferation was observed in HIV-infected viremic patients despite normal CD40L expression on activated CD4^+^ T cells. Reduced triggering of B cells by activated CD4^+^ T cells was clearly observed in HIV-infected viraemic patients and less obviously in aviraemic patients with comparable CD4^+^ T-cell counts [37]. This difference suggests that the B-cell dysfunction might be closely associated with the increase in virus replication. Massive efforts have been directed at identifying biomarkers to distinguish RPs from TPs, which are critical for early clinical intervention. It is technically easy to measure baseline B-cell counts and percentages in samples of whole blood. Moreover, sampling of whole blood is a practical and accessible way of detecting biomarkers. In our study, we found that the baseline B-cell count and percentage combination panel had a predictive accuracy of 80.7% for rapid disease progression, as measured by AUC. It suggests that the baseline B-cell count and percentage combination panel might be a candidate biomarker for the prediction of HIV disease progression.

### 4.1. The Limitations of the Study

Please note that the data presented in this study are preliminary. Since our cohort size was relatively modest and both TP and RP groups were small, more large-scale analyses and further evidence are needed to verify our results. All PHI subjects in the study were MSMs that were infected mostly by the CRF01_AE subtype of HIV. Whether our findings are applicable to HIV-infected individuals who received viral transmission through other routes and by other subtypes is difficult to know, and this will require confirmation from other studies.

## 5. Conclusion

Our findings shed new light on the impact of B-cell counts and percentages in early HIV infection as well as on the kinetic changes that B cells undergo during HIV infection. Our findings provide evidence that the changes in B-cell counts and percentages during PHI could predict disease progression in HIV-infected MSMs.

## Supplementary Material

The results shown in supplementary Figure 1, 2, and 3 are supplementary to the figures in the main paper. Supplementary Figure 1 was used to describe the percentages and absolute counts of B cells in RPs, TPs, and HIV-negative control groups at the baseline visit and the 12-month follow-up visit. Supplementary Figure 2 was used to display comparisons between CD4+ T-cell counts or HIV viral loads at the baseline visit and the 12-months follow-up visit in different groups based on the baseline B-cell counts. It indicates that the high absolute B-cell counts might be associated with the maintenance of high CD4+ T-cell counts. Supplementary Figure 3 was used to describe the association between the baseline counts/percentages of CD8+ T-cells and HIV disease progression. It suggests that the baseline CD8+ T-cell counts/percentages might be weak predictors of disease progression.

## Figures and Tables

**Table 1:** Difference between HIV-infected and HIV-negative individuals.

Characteristics	HIV^a^	HIV-negative^b^	*P* value
Gender male/female, number (%)	120 (100%)/0 (0%)	24 (100%)/0 (0%)	NS^c^
Age at first visit, year, median (IQR)	29 (17–64)	32 (23–45)	NS
Ethnicity, Han/no Han, number (%)	112 (93.3%)/8 (6.7%)	24 (100%)/0 (0%)	NS
HIV subtype, number (%)			
CRF01_AE	95 (79.2%)	—	
Other	20 (16.7%)	—	
Unknown	5 (4.1%)	—	
Fiebig stages^d^, number (%)			
I	0 (0.0%)	—	
II	19 (15.8%)	—	
III	0 (0.0%)	—	
IV	39 (32.5%)	—	
V	21 (17.5%)	—	
VI	41 (34.2%)	—	
Baseline HIV viral load, median log_10_ copies/mL (IQR)	4.71 (2.02–7.00)	—	
Baseline B-cell counts, cells/*μ*L, median (IQR)	123 (30–448)	257 (126–616)	<0.001
Baseline B-cell percentages, %, median (IQR)	6 (1–26)	10 (6–20)	<0.001
Baseline CD4^+^ T-cell counts, cells/*μ*L, median (IQR)	401 (87–1101)	748 (230–1682)	<0.001
Baseline CD8^+^ T-cell counts, cells/*μ*L, median (IQR)	1176 (407–23449)	622 (159–1280)	<0.001

All HIV-infected subjects were anti-HIV treatment naive at study entry. HIV-infected and healthy HIV negative individuals were compared using the chi-square test for categorical variables, independent *t*-test for normally distributed continuous variables, and the Mann–Whitney *U* test for non-normally distributed continuous variables.

^a^HIV, HIV-infected individuals.

^b^HIV negative, healthy HIV negative individuals.

^c^NS, not significant, *P* > 0.05.

^d^Fiebig stages was based on a combination of the estimated time post-infection at sampling and ancillary HIV PCR, Western blot and/or ELISA results.
