# Supplementary material for: Changes in B-Cell Counts and Percentages during Primary HIV Infection Associated with Disease Progression in HIV-Infected Men Who Have Sex with Men: A Preliminary Study
Source: Biomed Res Int. 2015 Sep 7;2015:468194. doi: 10.1155/2015/468194 (PMC4575999; doi:10.1155/2015/468194)
Supplement: Supplementary file 1 — The results shown in supplementary Figure 1, 2, and 3 are supplementary to the figures in the main paper. Supplementary Figure 1 was used to describe the percentages and absolute counts of B cells in RPs, TPs, and HIV-negative control groups at the baseline visit and the 12-month follow-up visit. Supplementary Figure 2 was used to display comparisons between CD4+ T-cell counts or HIV viral loads at the baseline visit and the 12-months follow-up visit in different groups based on the baseline B-cell counts. It indicates that the high absolute B-cell counts might be associated with the maintenance of high CD4+ T-cell counts. Supplementary Figure 3 was used to describe the association between the baseline counts/percentages of CD8+ T-cells and HIV disease progression. It suggests that the baseline CD8+ T-cell counts/percentages might be weak predictors of disease progression. [file 468194.f1.pdf]

Supplementary Materials

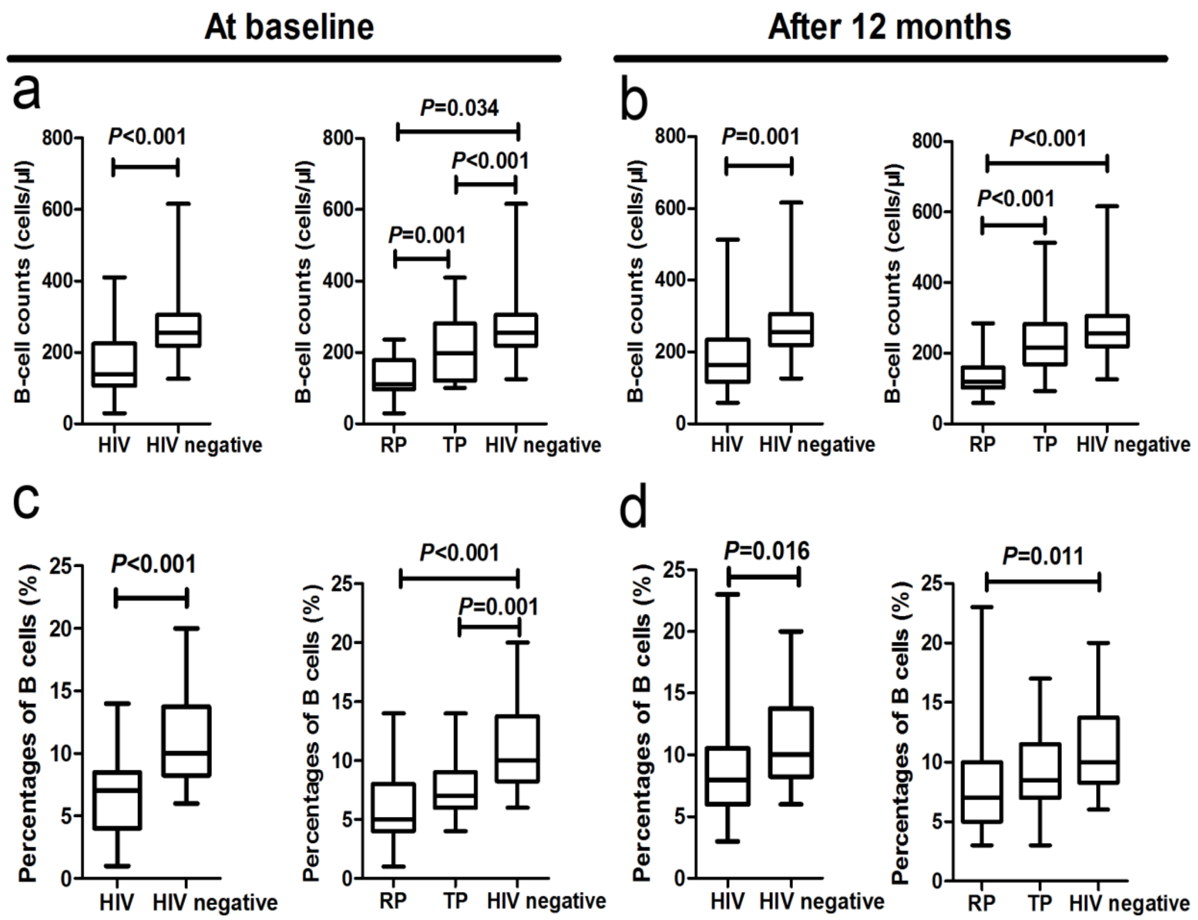

Supplementary Fig 1: The percentages and absolute counts of B cells in RPs, TPs, and HIV-negative control groups at the baseline visit and the 12-month follow-up visit. HIV represents HIV-infected subjects, including RPs and TPs. HIV negative represents the HIV-negative control group. Comparisons of B-cell counts (a) and percentages (c) at the baseline visit were made among the different groups. Comparisons of B-cell counts (b) and percentages (d) at the 12-month follow-up visit were made among the different groups. *P*-values $<$0.05 are considered statistically significant. The lines inside the boxes denote the medians.

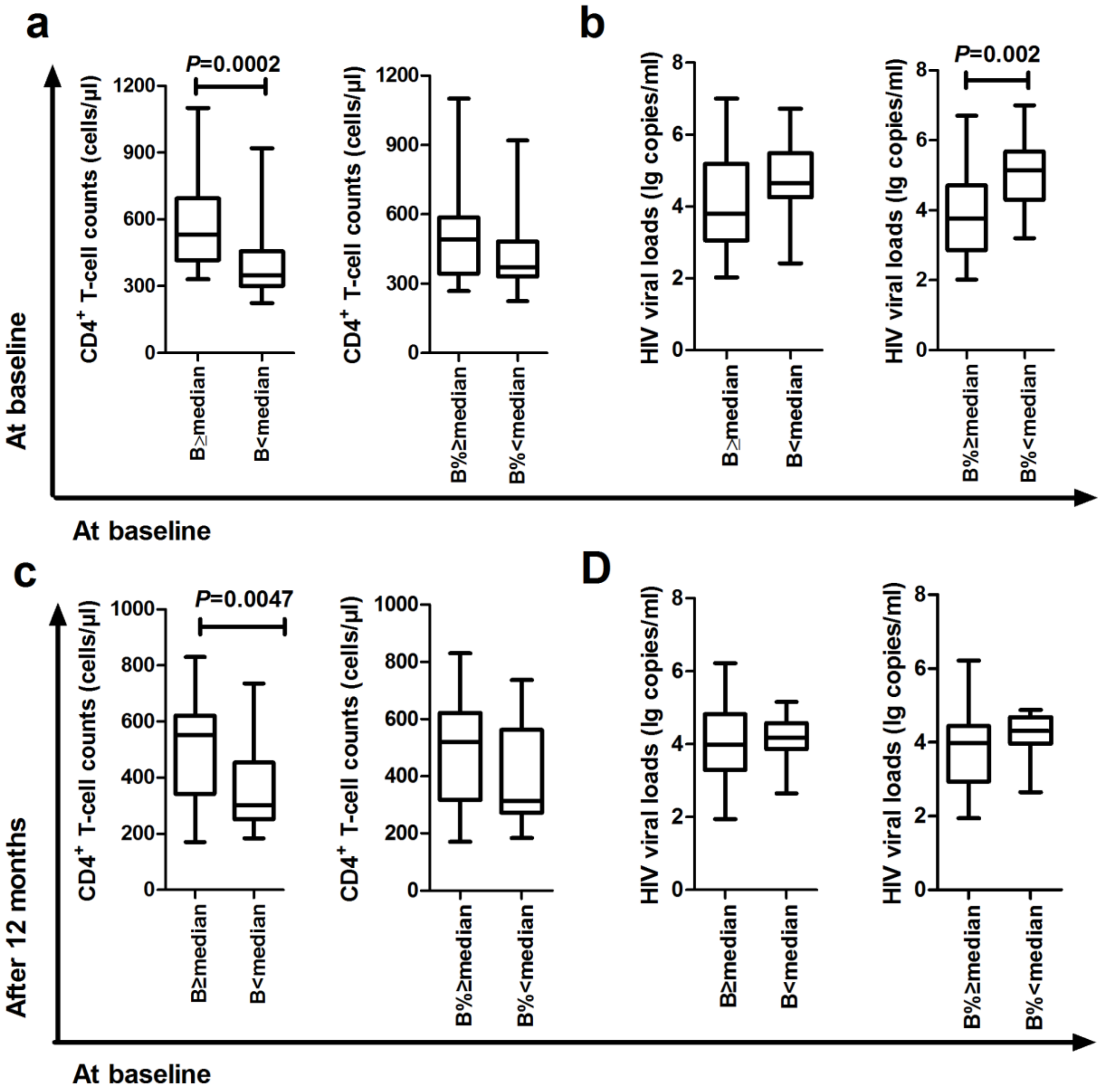

Supplementary Fig 2: Comparisons between $CD4^+$ T-cell counts or HIV viral loads at the baseline visit and the 12-months follow-up visit in different groups based on the baseline B-cell counts. We grouped 45 HIV-infected subjects based on the absolute B-cell counts or percentages at the baseline visit. Subjects were put into the ‘B ＜ median’ group when their absolute baseline B-cell counts were below the median (139 cells/μl). Alternatively, subjects were put into the ‘B ≥ median’ group when their absolute baseline B-cell counts were above or equal to the median. Similarly, our study subjects were also grouped into the ‘B% ＜ median’ group and the ‘B% ≥ median’ group based on how subjects’ baseline B-cell percentages were compared to the median (7%). Then we compared their $CD4^+$ T-cell counts or HIV viral loads at the baseline visit (a, b) and the 12-months follow-up visit (c, d). *P*-values＜0.05 are considered statistically significant. The lines inside the boxes denote the medians.

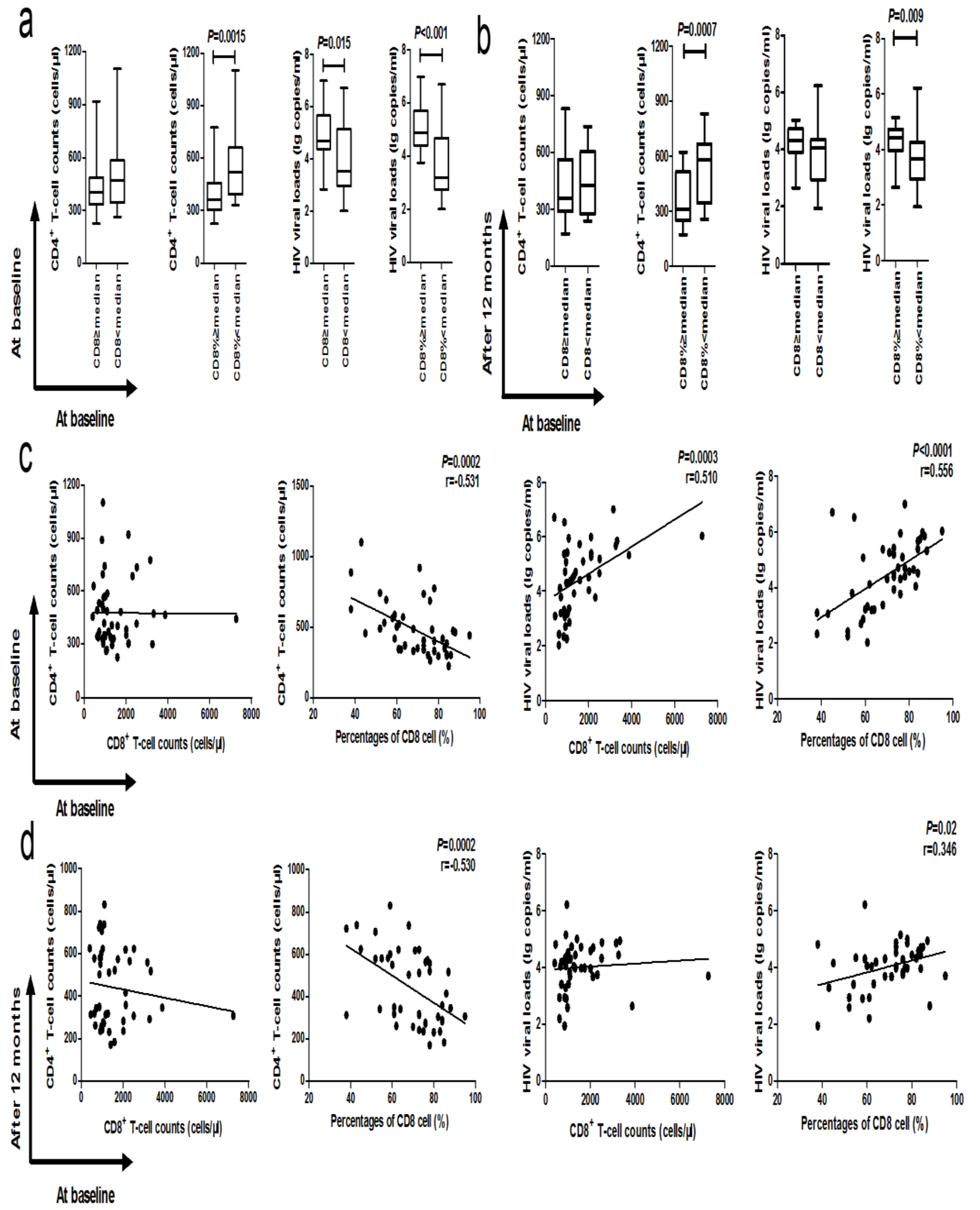

Supplementary Fig 3: Association between the baseline counts/percentages of CD8$^+$ T-cells and HIV disease progression. CD8$^+$ T-cell median counts and median percentages at the baseline visit were 1095 cells/μ l and 73%, respectively. According to these criteria, 23 subjects whose baseline CD8$^+$ T-cell counts were above 1095 cells/μ l belonged to the 'CD8 ≥ median' group, and 22 subjects whose baseline CD8$^+$ T-cell counts were below 1095 cells/μ l belonged to the 'CD8 < median' group. Among these individuals, 24 subjects whose baseline CD8$^+$ T-cell percentages were more than 73% were placed in the 'CD8% ≥

median' group, and 21 subjects whose baseline $CD8^+$ T-cell percentages were less than 73% were placed in the 'CD8% < median' group. (a) Comparisons of $CD4^+$ T-cell counts or viral loads at the baseline visit were made between different groups. The lines denote the medians. (b) Comparisons of $CD4^+$ T-cell counts or viral loads at the 12-month follow-up visit were made between the different groups. (c) Correlations between the baseline $CD8^+$ T-cells counts or percentages and the baseline $CD4^+$ T-cell counts or viral loads. (d) Correlations between $CD8^+$ T-cells counts/percentages at baseline visit and $CD4^+$ T-cell counts/viral loads at the 12-month follow-up visit. *P*-values <0.05 are considered statistically significant. The lines inside the boxes denote the medians.
